# Clinical insights of pregnancy management, adrenal insufficiency as a possible cause of elevated TSH: a pilot study of case series

**DOI:** 10.1186/s12884-023-06015-4

**Published:** 2023-10-04

**Authors:** Ken Kanazawa, Tatsuro Inaba, Shinichiro Koga, Koichiro Kuwabara

**Affiliations:** Department of Diabetes, Metabolism, and Endocrinology, Labor Health and Safety Organization, Tokyo Rosai Hospital, 4-13-21 Omoriminami, Ota-ku, 143- 0013 Tokyo, Japan

**Keywords:** Adrenal insufficiency, Hypothyroidism, Pregnancy management

## Abstract

**Background:**

The upper limit for thyroid-stimulating hormone has been strictly defined for pregnancy management, at which point levothyroxine replacement therapy will been initiated. However, it is essential to exclude adrenal insufficiency, including subclinical adrenal insufficiency, when initiating levothyroxine replacement therapy. However, in pregnancy management, it has rarely reported the incidence, clinical course, and characteristics of adrenal insufficiency as a possible cause of elevated thyroid-stimulating hormone.

**Methods:**

This case series study included pregnant patients undergoing thyroid-stimulating hormone management in a single-center diabetes endocrinology department between 2017 and 2020. The primary study outcome was the incidence of newly diagnosed adrenal insufficiency. We reported the clinical course and assessed the adrenal insufficiency characteristics at baseline and delivery and compared them with those of hypothyroidism.

**Result:**

Fifteen pregnant women were included for thyroid-stimulating hormone management; and nine were below the basal serum cortisol level, and four were newly diagnosed as having adrenal insufficiency (26.7%) with the endocrinological stimulation test. Among them, two cases exhibited nausea and hypoglycemic symptoms after the start of levothyroxine replacement therapy. In cases of adrenal insufficiency, the patients were successfully treated with appropriate steroid coverage.

**Conclusions:**

In the management of elevated thyroid-stimulating hormone levels during pregnancy, the frequency of adrenal insufficiency suspecting hypothyroidism may be higher than expected; therefore, we must be careful about starting levothyroxine replacement therapy for hypothyroidism. These clinical insights can have a significant impact on the pregnancy outcomes.

## Background

Adverse events in pregnancy have been reported to be associated with elevated thyroid-stimulating hormone (TSH) levels [1, 2]. Therefore, the 2017 American Thyroid Association (ATA) guidelines have strict upper threshold values for TSH levels and criteria for initiation of levothyroxine replacement therapy (LRT) for pregnancy management [3]. Although hypothyroidism (HT) is known to be the major cause of elevated TSH levels, it is important to exclude other causes of thyroid dysfunction such as pituitary tumors that secrete TSH, thyroid hormone resistance, and central hypothyroidism with biologically inactive TSH. One cause of elevated TSH is adrenal insufficiency (AI), which is a disorder characterized by the failure of adrenocortical function because of distorted function of hypothalamic-pituitary- adrenal (HPA) axis [4, 5], which is recognized as a rare condition, making early diagnosis of subclinical AI particularly difficult.

Previous case reports have described a case of subclinical AI in which the cause of elevated TSH was misdiagnosed as HT, and LRT was initiated, resulting in the manifestation of symptoms of AI [6, 7]. Therefore, during pregnancy, it is essential to exclude AI, including subclinical AI, when initiating LRT, following the ATA guidelines. However, in pregnancy management, the clinical course and characteristics of AI have rarely been reported as possible causes of elevated TSH levels.

In this pilot study we observed 15 patients over the course of TSH management during pregnancy, and we report the incidence, clinical course, and characteristics of newly diagnosed AI cases.

## Materials and methods

### Study design and participants, ethical considerations

This was a prospective case series. The study was conducted in an outpatient setting at the Department of Diabetes, Metabolism, and Endocrinology, Tokyo Rosai Hospital, Japan, from October 2017 to December 2020.

Participants were women who visited our institution for TSH management during pregnancy according to the following criteria. In women with no prior history of thyroid disorders, we used the reference range for TSH levels during pregnancy: TSH > 4 µIU/mL, according to the ATA guidelines. The other women had been initiated on LRT by the previous doctors, so we needed to reevaluate them carefully. In other words, we excluded hypothyroidism that existed before pregnancy. We selected a case in which hypothyroidism was suspected based on elevated TSH after pregnancy, and LRT was started according to the ATA guidelines by the previous doctors. We screened for the presence of HT or AI in the basal state, endocrinological basal value, thyroid ultrasound (TUS), and pituitary gland MRI. If the basal serum cortisol levels were less than the standard value, we suspected AI, performed an endocrinological stimulation test [8, 9], and diagnosed AI. The patients were then classified into the following groups and characterized (Group 1; AI group, Group 2; hypothyroid group with low basal serum cortisol but pass stimulation test, and Group 3; hypothyroid group with adequate basal serum cortisol).

This study adhered to the World Medical Association Declaration of Helsinki guidelines. The informed consent was obtained in oral form, with an option to opt-out from the study; moreover, the study protocol and opt-out were approved by the institutional review board of Tokyo Rosai Hospital (REC no. 02–32).

### Study outcomes

The primary study outcome was the incidence of newly diagnosed AI, including subclinical AI. The AI selection criteria had to satisfy all of the following conditions, from (1) to (3): (1) clinical symptoms or laboratory findings, such as acute general malaise, hypoglycemia, hypotension, weight loss, eosinophilia, and electrolyte abnormalities, that led to the suspicion of AI in outpatients. We focused on episodes of hypoadrenalism after LRT in patients with elevated TSH levels. (2) The basal serum cortisol level (collected between 6 and 8 a.m.) in the first, second, and third trimester of pregnancy was less than 10.8 µg/dL, 16.3 µg/dL, and 21.7 µg/dL, respectively [8, 9]. (3) A definitive diagnosis was made using the endocrinological stimulation test, standard-dose corticotropin stimulation test (SDST), low-dose corticotropin stimulation test (LDST), and corticotropin-releasing hormone stimulation test (CRH), on admission. SDST is a reliable dynamic test when primary AI is suspected during pregnancy. Measuring plasma 30 min-cortisol levels after 250 µg ACTH_1 − 24_ injection [1, 8, 9]. LDST, on the other hand, may be helpful in diagnosing secondary AI during pregnancy [10]. If the 30-min cortisol levels on SDST or LDST were less than 25.4, 29.0, and 32.6 µg/dL for the first, second, and third trimester of pregnancy, respectively [8–12], we diagnosed AI and performed glucocorticoid replacement therapy (GCRT). For GCRT in pregnant AI, we had selected hydrocortisone at a daily dose of typically 20–25 mg, mimicking the physiological cortisol secretion pattern [9]. Considering the gradual increase in both total and free cortisol during pregnancy, most women with AI had required to increase their daily dose of hydrocortisone during pregnancy. In this study, similar to the approach for nonpregnant patients, glucocorticoid replacement surveillance was predicated on clinical indicators, as no laboratory-based assessment has been definitively proven reliable. In other words, the appropriate hydrocortisone dosage was established on an outpatient basis, focusing on early morning cortisol levels for each stage and clinical symptoms and laboratory findings such as acute general malaise, hypoglycemia, hypotension, weight loss, eosinophilia, and electrolyte abnormalities [9].

The secondary study outcomes were [1] assessment of AI characteristics: endocrinological basal value, general blood sampling, and TUS at baseline; [2] comparison of AI and HT characteristics at baseline; and [3] comparison of AI and HT characteristics at the time of delivery after replacement therapy.

### Statistical analysis

Categorical variables are reported as raw frequencies (%). Continuous variables with normal and non-normal distributions were reported as mean ± standard deviation (SD) or median (interquartile range), respectively. We compared the two groups using Pearson’s chi-square test for categorical variables and Student’s *t*-test and Wilcoxon signed-rank test for normally and non-normally distributed continuous variables, respectively. All statistical analyses were performed using the JMP version 12 (SAS Institute Inc., Cary, NC, USA). Statistical significance was set at P < 0.05.

## Results

### Case summaries

In this study, we included 15 pregnant women for TSH management (Table 1), and will present a few representative cases.

**Table 1:** the patients’ baseline characteristics

Case	Basal state				Endocrinological basal value						TUS		pituitary gland MRI
	Pregnacy (weeks)	Age (yr)	Symptoms	LT4 (µg/Day)	TSH (µIU/mL)	FT4 (ng/dL)	TgAb (U/mL)	TPOAb (U/mL)	BC (µg/dL)	ACTH (µg/dL)	Internal properties	Estimated weight (g)	
**Group 1; AI group (n = 4)**													
1	11	34	none	25	2.6	0.9	45.6	14.7	6	28.9	DP	33.6	NA
2	14	42	nausea	none	4.88	0.96	≦ 10	5	10.5	5.7	NP	6.6	NA
3	15	33	none	25	1.46	1.44	1.24	1.28	10.7	22.5	NP	none	Ratke cyst
4	17	31	nausea	37.5	0.81	1.04	5.1	<0.50	15.3	14.8	DP	13.1	none
**Group 2; hypothyroid group with low basal serum cortisol but pass stimulation test (n = 3)**													
5	7	22	fatigue	none	10.8	0.96	183	1450	8	10.4	DP	20.3	NA
6	12	31	nausea	none	5.73	1.09	2.93	<0.50	12	18.9	NP	8.1	pituitary cyst
7	15	37	none	75	1.17	1.21	≦ 10	12.43	10.4	10.8	NP	5.5	NA
**Group 3; Hypothyroid group with adequate basal serum cortisol (n = 6)**													
8	14	41	nausea	50	1.2	0.99	0.78	<0.50	11.1	9	NP	8.8	none
9	16	24	none	50	2.96	1.34	1.2	26.5	24.8	none	DP	7.9	none
10	21	31	mild	none	5.6	0.85	≦ 10	10	22.3	14.7	NP	9.5	none
11	27	34	none	none	5.57	0.79	0.8	1.17	24.5	16.9	NP	7.2	none
12	31	35	nausea	50	1.73	0.9	0.45	<0.50	27.6	12.6	NP	7.8	none
13	33	28	nausea	37.5	2.31	1.11	1.96	<0.50	21.3	9.3	NP	7.1	none
**low basal serum cortisol but refused stimulation test (n = 2)**													
14	19	29	hypoglyceimia	12.5	1.8	1.08	11.5	0.61	13.4	6.3	NP	8.1	NA
15	31	34	nausea	100	0.59	1.13	88	281	16.8	none	NP	9.7	none

TUS, Thyroid Ultrasound; TD, Thyroid Disease; LT4, Levothyroxine;

TSH, Thyroid‑stimulating hormone; TgAb, thyroglobulin autoantibodies;

TPOAb, thyroid peroxidase antibodies; BC, Basal Cortisol; AI, Adrenal Insufficiency;

DP, diffuse thyroid parenchyma; NP, normal thyroid parenchyma;NA, No abnormality

#### Case 2

This patient was a 42-year-old Asian woman with a 14-week pregnancy. She visited our hospital with a chief complaint of nausea and elevated TSH (4.88 µIU/mL). Her medical history revealed no thyroid or other endocrine disease. Additionally, she was a healthy, nonsmoking, iodine-sufficient woman with a balanced diet. TUS showed no abnormal findings, thyroglobulin autoantibodies (TgAb) were negative, and thyroid peroxidase antibodies (TPOAb) were mildly positive (5 U/mL); therefore, we considered LRT. However, basal serum cortisol and ACTH were low (10.5 µg/dL and 5.7 µg/dL), LDST, SDST, and CRH were unresponsive, and pituitary MRI showed mild enlargement of the anterior lobe. We therefore diagnosed AI and initiated GCRT instead of LRT. After replacement therapy, her TSH level returned to almost within the normal limits.

#### Case 3

This patient was a 33-year-old Asian woman with a 15-week pregnancy. At 10 weeks, the previous physician found elevated TSH levels, and mildly positive TPOAb, and initiated LRT. We found low basal serum cortisol (10.7 µg/dL), suspected a relative AI associated with LRT, and withdrew levothyroxine (LT4). LDST and CRH were unresponsive, and pituitary MRI revealed a Rathke cyst. We diagnosed the patient with AI and initiated GCRT. After replacement therapy, her TSH level returned to almost within the normal limits.

### The incidence of newly diagnosed AI, comparison of baseline characteristics

**Fig. 1:**
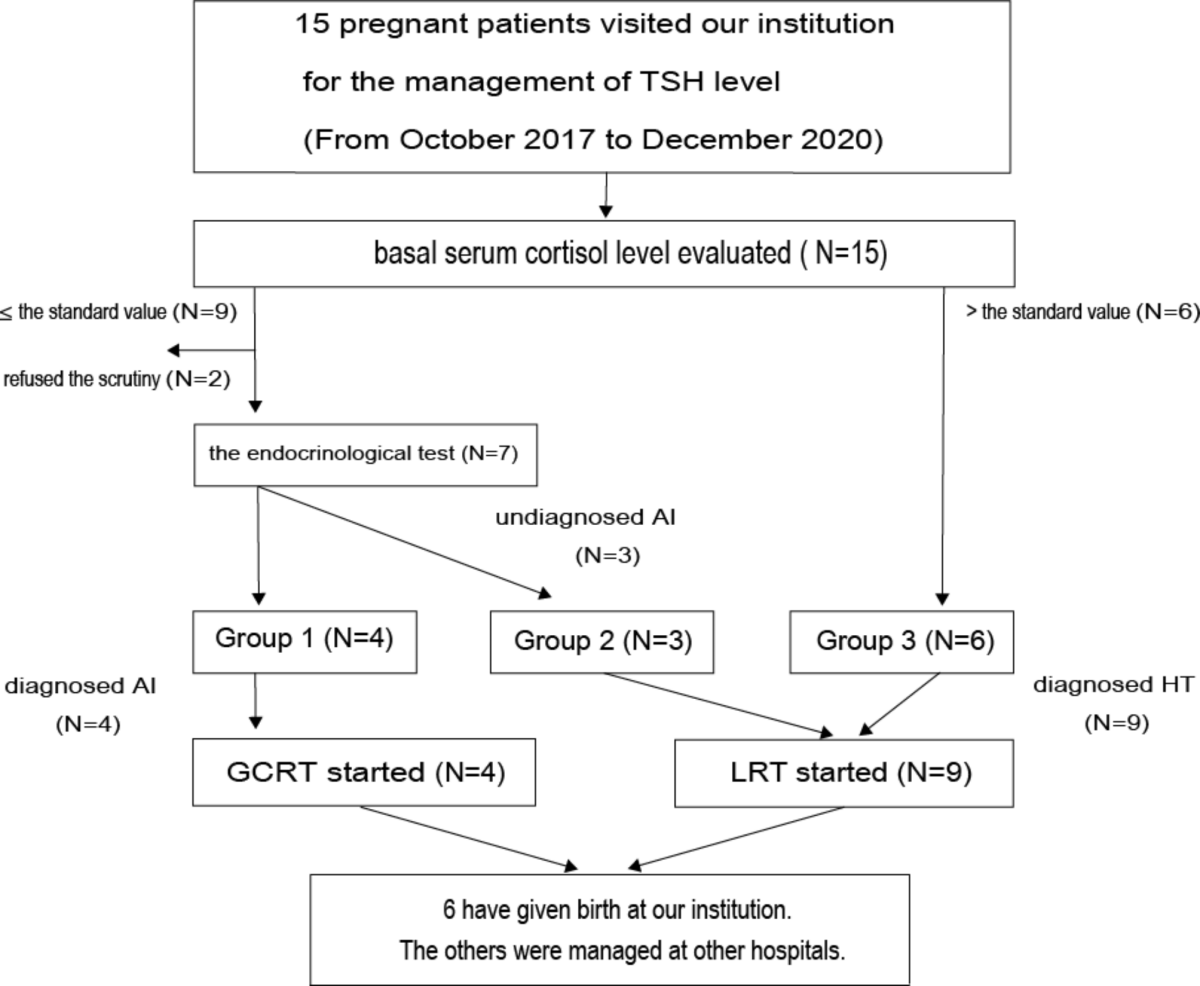
Flow diagram showing enrollment and follow-up of the study participants. Group 1; AI group, Group 2; hypothyroid group with low basal serum cortisol but pass stimulation test, Group 3; hypothyroid group with adequate basal serum cortisol. TSH, thyroid-stimulating hormone; AI, adrenal insufficiency; HT, hypothyroidism; GCRT, glucocorticoid replacement therapy; LRT, levothyroxine (LT4) replace therapy

**Fig. 2:**
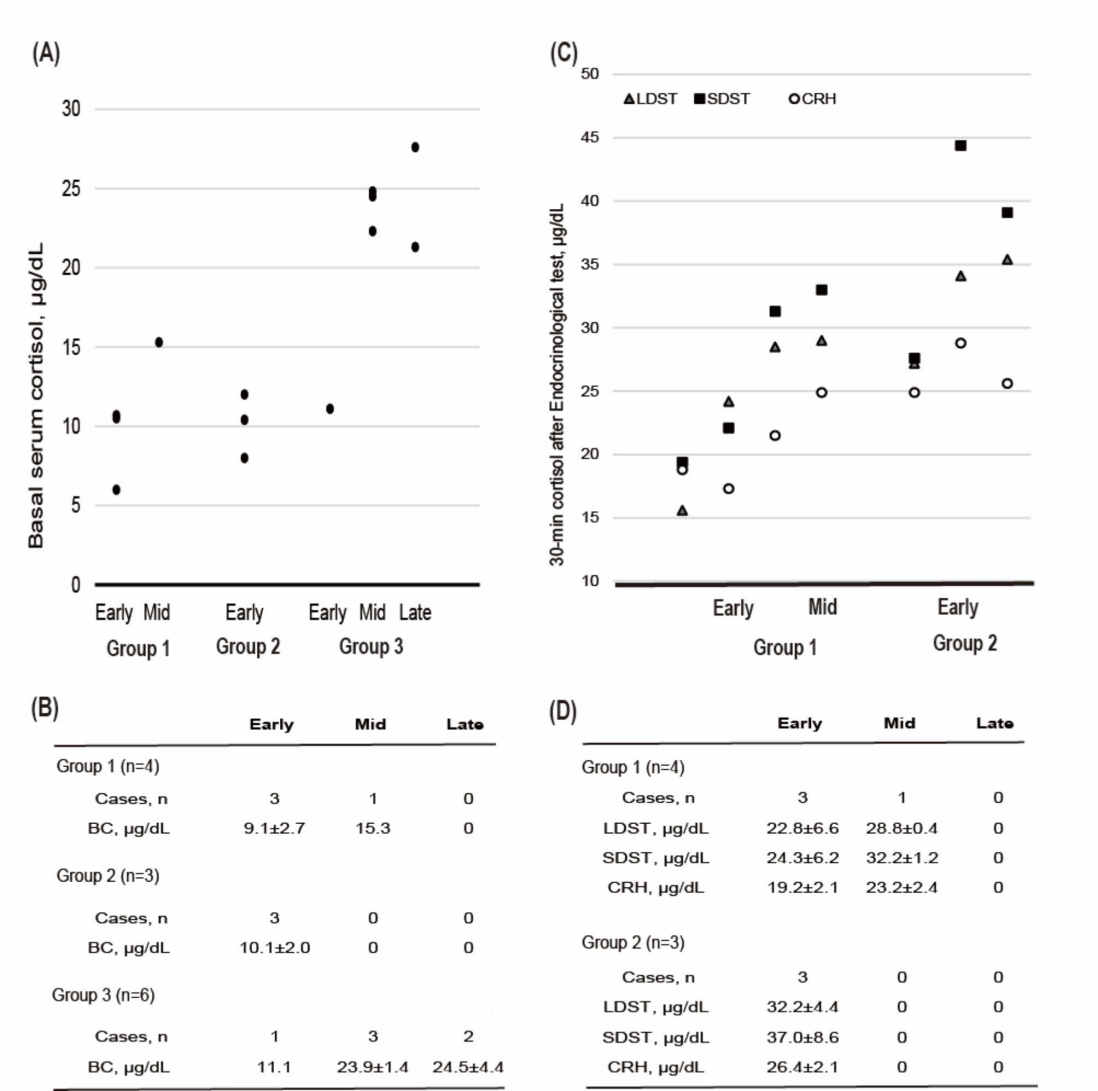
Results of the basal serum cortisol levels and endocrinological stimulation test. (**A**) Basal serum cortisol levels (Group1, 2, and 3) in each case. (**B**) Mean basal serum cortisol levels (Group1, 2, and 3) during pregnancy. (**C**) Endocrinological stimulation test results (LDST, SDST, and CRH) the figure indicates 30-min cortisol levels after each stress. (**D**) Mean 30-min cortisol levels after each stress in the pregnancy stage. HT, hypothyroidism; AI, adrenal insufficiency; early, early pregnancy (1~15w); mid, mid-pregnancy (16 ~ 27w); late, late pregnancy (28w~). Group 1; AI group, Group 2; hypothyroid group with low basal serum cortisol but pass stimulation test, Group 3; hypothyroid group with adequate basal serum cortisol. BC, basal cortisol; LDST, low-dose corticotropin stimulation test; SDST, standard-dose corticotropin stimulation test; CRH, corticotropin-releasing hormone stimulation test

As a result, nine pregnant women had less than the basal serum cortisol levels in the first and second trimesters of pregnancy (Fig. 1). With the endocrinological stimulation test on admission, four pregnant women were newly diagnosed with AI (Group 1; AI group, 26.7%), three were not diagnosed (Group 2; hypothyroid group with low basal serum cortisol but pass stimulation test), and two refused the examination; six were classified as HT (Group 3; Hypothyroid group with adequate basal serum cortisol). Figure 2 shows the results of the basal serum cortisol level and endocrinological stimulation test, in the stage of pregnancy. The mean basal serum cortisol levels in the Group 1 were 9.1 ± 2.7 µg/dL, 15.3 µg/dL, those for Group 2 was 10.1 ± 2.0 µg/dL, and Group 3 were 11.1 µg/dL, 23.9 ± 1.4 µg/dL, 24.5 ± 4.4 µg/dL, in the first, second, and third trimester respectively. In Group 1, the mean 30-min cortisol levels after LDST were 22.8 ± 6.6 µg/dL, 28.8 ± 0.4 µg/dl, SDST were 24.3 ± 1.9 µg/dL, 32.2 ± 1.2 µg/dL, and CRH were 19.2 ± 2.1 µg/dL, 23.2 ± 2.4 µg/dL, first trimester, and second trimester, respectively. Table 2 shows the clinical characteristics of AI compared to HT, examining both Group 1 vs. Group 3 and Group 1 vs. Group 2. The eosinophil counts for Group 1 was found to be 2.9 ± 1.1%, while Group 2 had an average of 1.3 ± 0.9%, and Group 3 had an average of 1.0 ± 0.5%. The differences in eosinophil counts among the groups were statistically significant (Group 1 vs. Group 2, P = 0.0215; Group 1 vs. Group 3, P = 0.0043), as determined by the student’s t-test. However, the difference in eosinophil percentages between the groups is not clinically meaningful. Although the p-value indicated statistical significance, it is challenging to derive clinical relevance due to the small sample and difference. The TPOAb, TgAb, and TUS findings (diffuse thyroid parenchyma and estimated weight) were not significantly different.

**Table 2:** Comparison of baseline characteristics between AI and HT

	**Group 1**			Group 2		Group 3				P-value	
	**Total**	Early	Mid	**Total**	Early	**Total**	Early	Mid	Late	1 vs 2*	1 vs 3*
**Cases, n**	4	3	1	3	3	6	1	3	2		
**Variables**											
**Pregnacy, weeks**	14.3 ± 2.5	13.3 ± 2.1	17		11.3 ± 4	23.7 ± 7.9	14	21.3 ± 5.5	32 ± 1.4	n. s.	n. s.
**Age, yr**	35.0 ± 4.8	36.3 ± 4.9	31		30.0 ± 7.5	32.2 ± 5.9	41	29.7 ± 5.1	31.5 ± 4.9	n. s.	n. s.
**BMI, kg/m** ^**2**^	27.2 ± 6.9	28.2 ± 8.1	24.2		19.2 ± 2.1	23.5 ± 4.0	22.8	21.5 ± 2.9	26.8 ± 5.4	n. s.	n. s.
**WBC, ×10** ^**2**^ **/µL**	81.8 ± 16.5	80.3 ± 19.9	86		74.3 ± 19.1	80.2 ± 16.4	80	77 ± 12.2	85 ± 31.1	n. s.	n. s.
**Eosino, %**	2.9 ± 1.1	2.4 ± 0.6	4.4		1.3 ± 0.9	1.0 ± 0.5	0.8	1.1 ± 0.8	1	0.0215	0.0043
**Hb, g/dL**	11.4 ± 0.5	11.7 ± 0.3	10.8		11.5 ± 0.8	11.6 ± 0.9	11.9	11.6 ± 1.4	11.6 ± 0.2	n. s.	n. s.
**Na, mEq/L**	136.7 ± 1.0	136.6 ± 1.2	137		138 ± 1.0	138.3 ± 1.5	137	138 ± 1.7	139.5 ± 0.7	n. s.	n. s.
**K, mEq/L**	4.1 ± 0.2	4.1 ± 0.2	4		4.2 ± 0.3	3.8 ± 0.3	3.8	3.5 ± 0.5	3.9 ± 0.1	n. s.	n. s.
**LDL-C, mg/dL**	106.8 ± 14.0	100 ± 7.9	127		75 ± 29.8	128.8 ± 22.2	117	119.3 ± 9.7	149 ± 32.5	n. s.	n. s.
**TG, mg/dL**	159.8 ± 62.1	130 ± 19.1	81		108 ± 12.5	199.5 ± 70.9	336	184.7 ± 28.1	153.5 ± 4.9	n. s.	n. s.
**HbA1c, %**	5.2 ± 0.26	5.2 ± 0.3	4.9		5.2 ± 0.3	5.4 ± 0.3	5.7	5.2	5.5 ± 0.4	n. s.	n. s.
**TSH, µIU/mL**	2.4 ± 1.8	2.98 ± 1.7	0.81		5.9 ± 4.8	3.2 ± 1.9	1.2	4.7 ± 1.5	2.0 ± 0.4	n. s.	n. s.
**FT4, ng/dL**	1.1 ± 0.2	1.1 ± 0.3	1.1		1.1 ± 0.1	1.0 ± 0.2	0.99	1.0 ± 0.3	1.0 ± 0.1	n. s.	n. s.
**TgAb, U/mL**	7.6 (2.2, 36.7)	10 (1.2, 45.6)	5.1		92.97 (2.9, 183)	1.1 (0.7, 4.0)	0.78	1.3 (0.8, 10)	1.2 (0.5, 2.0)	n. s.	n. s.
**TPOAb, U/mL**	3.14 (0.7, 12.3)	5 (1.3, 14.7)	0.5		12.4 (0.5,1450)	0.8 (0.5, 3.4)	0.5	1.2 (1.2, 10)	0.5	n. s.	n. s.
**ACTH, µg/dL**	17.9 ± 10.0	19.0 ± 12	14.8		13.4 ± 4.8	14.6 ± 5.9	9	18.8 ± 5.3	26.8 ± 5.4	n. s.	n. s.
**TUS**											
**diffuse thyroid ** **parenchyma**, **n (%)**	2(50)	1(33.3)	1(100)		1(33.3)	1(16.7)	0(0)	1(33.3)	0(0)	n. s.	n. s.
**Estimated weight, g**	13.3 ± 14.5	20.1 ± 19.1	13.1		11.3 ± 7.9	8.0 ± 0.9	8.8	8.2 ± 1.2	7.4 ± 0.4	n. s.	n. s.

AI, adrenal insufficiency; HT, hypothyroidism

Group 1; AI group, Group 2; hypothyroid group with low basal serum cortisol but pass stimulation test, Group 3; hypothyroid group with adequate basal serum cortisol. Early, early pregnancy (1~15w); Mid, mid-pregnancy (16 ~ 27w); Late, late pregnancy (28w~)

BMI, body mass index; ALB, albumin; LDL-C, LDL cholesterol; TG, Triglyceride; HbA1c, hemoglobin A1c; TgAb, thyroglobulin autoantibodies; TPOAb, thyroid peroxidase antibodies; BC, Basal Cortisol; TUS, Thyroid Ultrasound; n. s., not significant

Continuous and categorical data are expressed as the mean ± standard deviation or median (interquartile range) and number (%), respectively

*Comparison of values between patients (Group 1 vs. Group 2 and Group 1 vs. Group 3) using Pearson’s chi-square test for categorical variables and using Student’s t-test and the Wilcoxon signed-rank test for normally distributed and skewed continuous variables, respectively

### Pregnancy outcome after replacement therapy

In this study, two AI patients who were started on GCRT were followed up until delivery. Table 3 shows the clinical delivery characteristics of AI compared to HT; gestational age (wk), mode of delivery, fetal sex, weight, height, Apgar scores, and placenta weight were not statistically significantly different. In the AI group, two emergency caesarean sections were required, one for non-reassuring fetal status, the other for intrauterine infection.

**Table 3:** Comparison of AI and HT characteristics at the delivery after replace therapy

	AI (n = 2)	HT (n = 4)	P-value^*^
Gestational age (wk)	38.5 ± 0.70	40.3 ± 1.0	n. s.
Mode of delivery			0.067
ND, n (%)	0 (0)	4 (100)	
CS, n (%)	0 (0)	0 (0)	
ECS, n (%)	2 (100)	0 (0)	
Male gender, n (%)	1 (50)	2 (50)	n. s.
Birth weight (g)	2530.5 ± 120.9	3038.8 ± 350.3	n. s.
Birth height (cm)	46.7 ± 1.9	48.9 ± 2.0	n. s.
1-min Apgar	8.0	8.8 ± 0.5	n. s.
5-min Apgar	9.0	9.5 ± 0.6	n. s.
Placenta weight (g)	472.0 ± 82.0	580.0 ± 88.3	n. s.

AI, adrenal insufficiency; HT, hypothyroidism

ND, normal delivery; CS, Caesarean Section: ECS, Emergency Caesarean Section

n. s., not significant

*Comparison of values between patients with AI and HT

Continuous and categorical data are expressed as the mean ± standard deviation or median (interquartile range) and number (%), respectively

Then, one of the four patients diagnosed with AI during pregnancy were reevaluated in an endocrinologic study. The basal serum cortisol and ACTH levels were 10.9 µg/dL, 6.7 pg/mL, respectively. The 30-min cortisol levels after LDST were 17.8 µg/dL; SDST levels were 22.9 µg/dL; CRH levels were 17.6 µg/dL; and ITT levels were 20.3 µg/dL, respectively. Finally, one patient was diagnosed with secondary AI and continued GCRT.

## Discussion

In this prospective case series, we identified two important clinical issues. First, the incidence of AI-suspected HT may be higher than expected during pregnancy. Fifteen pregnant women were suspected of having HT based on findings such as elevated TSH and four were newly diagnosed with AI (26.7%). Pregnancy affects the maternal HPA-axis, leading to an increased placental production of estrogen. This stimulates hepatic corticosteroid-binding globulin (CBG) production, thus stimulating cortisol production [13, 14]. Total plasma cortisol, 24-h urine free cortisol and CBG levels progressively rise threefold during pregnancy [13]. Third trimester plasma cortisol varies over a wide range, from 16.3 to 55 µg/dl, and decreases promptly after delivery [15]. Plasma ACTH levels rise through pregnancy, which parallel the rise in cortisol, reaching maximal levels during labor and delivery. The cause of this increase in ACTH is not clear, but placentally derived ACTH may be a significant contributor to hypercortisolism in pregnancy [16, 17]. Other causes may include pituitary desensitization to cortisol feedback, or enhanced pituitary responses to corticotropin-releasing factors such as vasopressin and CRH [13]. Therefore, it is not easy to make conclusions about the level of cortisol, ACTH, CRH in pregnant women, especially to confirm the presence of a subclinical AI. The causes of AI have been extensively reviewed. It is important to distinguish primary AI from Sheehan syndrome with anterior pituitary hormone deficiency or other secondary causes [13]. However, ACTH levels and the CRH stimulation test, which is useful in differentiating tertiary from secondary AI in nonpregnant individuals, has only limited utility in pregnancy [18]. Thus, it is difficult to determine at what level and to what degree dysfunction of the HPA axis occurs in pregnant women. Recent data have reported an AI prevalence as low as 144 per 1 million people; therefore, we first suspected hypothyroidism based on findings such as elevated TSH. However, as noted above, the diagnosis of subclinical AI is difficult, and the prevalence of subclinical AI is unknown in the complex HPA axis changes during pregnancy. Therefore, we must be aware of AI and its manifestation due to various pregnancy-related stresses. In particular, labor, vaginal delivery, and cesarean section are acutely stressful situations, and CRH, ACTH, and cortisol levels increase several-fold with the onset of labor and delivery [19–21]. Therefore, it is important to consider subclinical AI and take appropriate action during stress. In this study, two newly diagnosed AI were successfully delivered with appropriate steroid coverage.

Second, subclinical AI may be included as a cause of elevated TSH; therefore, we must exercise caution when starting LRT for HT. Previous studies have shown that AI is associated with maternal mortality in pregnancy [22, 23], and further AI exacerbation due to the inadvertent start of LRT must be avoided. Many studies have shown that HT causes adverse events during pregnancy, and TPOAb status has an additional adverse impact. In addition, the treatment of TPOAb-positive women with TSH > 2.5mU/L resulted in a significant reduction in pregnancy complications [24]. Therefore, the ATA guidelines recommend that if TPOAb is positive and TSH is greater than the pregnancy-specific reference range, LRT should be initiated [3, 25]. However, in this study, two cases showed nausea and hypoglycemic symptoms after the start of LRT. In the AI group, the basal serum cortisol level was less than the standard value. This clinical finding makes AI suspicious [23], and an endocrinologic stimulation test should be performed whenever possible to differentiate it from HT [23]. There was no difference in the TPOAb and TUS findings between the groups. Additionally, in severe AI with mildly elevated TSH, vigilant monitoring is necessary due to the expected compensatory decrease in free T4. Therefore, when initiating LRT in pregnancy with elevated TSH levels, it is important to monitor basal serum cortisol, clinical symptoms after administration, in addition to TPOAb and TUS findings.

We have observed two patients diagnosed with AI, who experienced unfavorable pregnancy outcomes: intrauterine infection and non-reassuring fetal status following GCRT. It is widely recognized that these outcomes may be associated with improper glucocorticoid use. Indeed, the appropriate dosage of hydrocortisone is challenging to determine during pregnancy. This is due to the overlap of symptoms between over- and under-replacement of glucocorticoids and common pregnancy [9]. The exact relationship between these outcomes and glucocorticoid usage in this study remains unclear. Nevertheless, given the potential risks, it is important to ensure meticulous prescribing and monitoring of patients to prevent both over- and under-dosing of glucocorticoids [22].

### Limitations of the study

This study has a few limitations. First, it was a single-center study conducted in one diabetes endocrinology department. Thus, the sample size was small, and these results may lack generalizability and evidence. Future prospective multicenter trials with sufficient sample sizes are needed to prove AI incidence. Second, the patients who dropped out might have had different outcomes. Finally, there is a lack of consensus regarding the interpretation of endocrinological measures and stimulation tests for AI diagnosis in pregnancy. Because it is not easy to draw conclusions about the evaluation of the HPA-axis during pregnancy. In this study, we referenced the results of basal cortisol levels and SDST, as discussed in previous studies [8, 9, 17]. In order to distinguish between AI and HT, we require not only basal cortisol levels but also interpretation of the stimulus test, as in Group 2 of this study. However, as for the stimulation studies, ACTH_1 − 24_ and CRH (human) are licensed by the FDA as category C drugs for administration during pregnancy only when there is a clear indication. Therefore, in addition to stimulation testing, we must better understand the interpretation of other more convenient endocrinological measures for the diagnosis of AI. Indeed, this study required a confirmatory evaluation of 24-hour urinary free cortisol, CBG levels, ACTH, and estrogenic effects during pregnancy and AI after delivery. Therefore, future studies should discuss specific interpretations of more convenient endocrinological measures, LDST, and CRH results during pregnancy.

## Conclusions

In the management of TSH in pregnancy, the incidence of AI being misdiagnosed as HT may be higher than expected; therefore, we must be careful about initiating LRT for HT. AI diagnosis is uncertain and difficult, especially with the interpretation of endocrinological stimulation tests. However, it is necessary to consider the possibility of AI, which can have a significant impact on pregnancy, so this case series offers clinically interesting insights.

## Data Availability

The datasets generated and/or analyzed during the current study are not publicly available to protect participants’ privacy but are available from the corresponding author upon reasonable request.

## Abbreviations

AI: Adrenal insufficiency
HPA: Hypothalamic–pituitary–adrenal
TSH: Thyroid‑stimulating hormone
LRT: Levothyroxine replacement therapy
HT: Hypothyroidism
ATA: American Thyroid Association
TUS: Thyroid Ultrasound
SDST: Standard-dose corticotropin stimulation test
LDST: Low-dose corticotropin stimulation test
CRH: Corticotropin-releasing hormone stimulation test
GCRT: Glucocorticoids replace therapy
SD: Standard deviation
TgAb: Thyroglobulin autoantibodies
TPOAb: Thyroid peroxidase antibodies
LT4: Levothyroxine
CBG: Corticosteroid-binding globulin
